# Addressing Future Epidemics: Historical Human Rights Lessons from the AIDS Pandemic

**DOI:** 10.20411/pai.v1i1.60

**Published:** 2016-05-20

**Authors:** Ambar Mehta, Thomas C. Quinn

**Affiliations:** 1 Johns Hopkins University School of Medicine, Baltimore, Maryland; 2 Johns Hopkins University Center for Global Health, Baltimore, Maryland; 3 Division of Intramural Research, National Institute of Allergy and Infectious Diseases, NIH, Bethesda, Maryland

**Keywords:** HIV/AIDS, Ebola, Public health, Epidemics, Human rights, United States, India

## Abstract

**Introduction:**

The Ebola epidemic in West Africa sparked many ethical and polarizing public health questions on how to adequately control transmission of the virus. These deliberations had and will continue to influence patients, healthcare workers, public perceptions of disease, and governmental responses. Such extensive and potential ramifications warranted an analysis of prior epidemics to sufficiently inform policy makers and prepare them and other authorities for future epidemics. We analyzed how the general public, medical institutions, federal government, and patients themselves responded during the early stages of the AIDS pandemic in two different countries and cultures, the United States and India.

**Discussion:**

Our analysis identified four key findings pertaining to the human rights of patients and healthcare workers and to the crucial roles of the government and medical community. The first demands that authoritative officials acknowledge the presence of high-risk behaviors and properly educate the public without stigmatizing groups of individuals. For this task, the medical community and federal government must form and display to the public a respectful and collaborative partnership towards battling the epidemic. These two synergistic endeavors will then allow appropriate officials to implement effective, yet civil, interventions for limiting transmission. Finally, the same officials must ensure that their interventions maintain the human rights of high-risk populations and of healthcare workers.

**Conclusions:**

Applying these findings to future epidemics of infectious diseases can aid policy makers in navigating complicated ethical and public health questions, and help prevent them from repeating past mistakes in handling epidemics.

Understanding human rights lessons from the early AIDS pandemic can aid policy makers in addressing future epidemics of infectious diseases.

## INTRODUCTION

West Africa's Ebola epidemic raised countless ethical and complex public health questions [1]. Many debated the merits surrounding mandatory quarantines of healthcare professionals, the patient's autonomy during a crisis, the roles of governments, and the implementation of travel bans [2,3]. Such significant deliberations warrant a review of prior epidemics. Specifically, investigating how societies and governments responded to a past epidemic can highlight (1) the basic human rights that must be upheld during an epidemic, and (2) the irrevocable roles of involved parties, such as the federal government and the medical community. Policy makers must incorporate these lessons when discussing the aforementioned public health questions to prevent repeating mistakes from the past.

Towards these goals, physicians, policy makers, researchers, and historians can study the early AIDS pandemic, as it shares many similarities with the current Ebola epidemic. The pathologic agent in each disease is a virus that spreads through bodily fluids [4]. Transmission in both is associated with common and often stigmatizing human behaviors [4,5]. While Ebola affects the patient acutely and AIDS can become a chronic illness, both are fatal without treatment and originated in Africa [4]. Many characteristics that create the cultural, structural, and economic fabric of our societies, such as fear, stigma, and victim blaming, have influenced responses in both epidemics [6].

We examined the early AIDS epidemic in the United States and India, and specifically investigated the interplay between the general public, federal government, medical community, and patients. Identifying common lessons of an epidemic that pertain to two countries that differ vastly in culture, socioeconomic status, and political atmosphere, helps elucidate indispensable and generalizable lessons for future outbreaks of infectious diseases.

## UNITED STATES

During the early 1980s, a group of previously healthy homosexual men in California and New York perplexed physicians when they presented with Kaposi's sarcoma, a rare form of cancer, and numerous opportunistic infections [7,8]. Scientists initially believed that these patients obtained their illnesses through sexual behavior and linked the disease to homosexual activity [9]. When further research uncovered that a severely weakened immune system constituted the underlying pathology in these patients, scientists coined the name GRID, or gay-related immune deficiency [10]. Soon, researchers identified other susceptible groups and informally created the “4H club” for those at greater risk for acquiring the disease—homosexual men, hemophiliacs, heroin users, and Haitians [11]. To reflect the underlying immune state, the name of the disease eventually changed to AIDS.

Regarding hemophiliacs, they developed their own epidemic of HIV because of their need for blood products [12]. One study described that an astonishing 92% of individuals with hemophilia A had been exposed to HIV [13]. Only after the discovery of the responsible virus, the development of appropriate assays, detection of infected donors, and the inactivation of virus in antihemophilic factor preparations was the transmission rate among hemophiliacs significantly reduced [13].

Discrimination and stigma from the general public swiftly inundated AIDS patients [14]. Many believed that the disease served as punishment for the homosexual community and injection drug users. Others claimed that God reprimanded Haitians with the virus because they were foreigners and were involved in the same risky behaviors. Isolation also reached hemophiliacs, who required blood transfusions for their survival; the most notable story involved a middle school student, Ryan White, who was expelled from school after he received an HIV-contaminated blood transfusion [15].

Overall, the American public associated the disease with affected groups of individuals that they considered a minority and “undesirables.” Common misconceptions, such as becoming infected from occupying the same room as an HIV-positive individual, physical contact, or being bitten by a mosquito that had previously bitten an infected individual, fueled this stigmatizing sentiment [16]. Consequently, these sentiments led to many ineffective attempts to reduce transmission of the virus. One study described how negative feelings towards people with AIDS, such as feeling “angry”, “afraid,” or “disgusted,” led to greater support for punitive measures such as mandatory quarantines [17]. History has shown that these compulsory public health measures have not been effective in controlling epidemics [18]. For example, the aggressive response to the HIV epidemic in Cuba during the 1980s and 1990s, which largely involved mandatory quarantines, inhibited appropriate public health measures from taking place sooner [19,20].

This sentiment also partially explains why the government did little to address the early epidemic; President Reagan only mentioned AIDS publicly for the first time in 1985, four years after its recognition [21]. Furthermore, he refused to endorse safer sex and contraception as methods for reducing transmission [22]. Instead, he suggested practicing sexual abstinence and contemplated banning HIV-positive immigrants from entering the country [23]. These quixotic viewpoints illustrated an overt denial toward the prevalence of high-risk behaviors in the country and, more importantly, a conscious disregard for and apathy toward those whom most believed were at risk for infection. In summary, the government and public abhorred the disease, but more so the behaviors associated with it; little was done to stop virus transmission or to broadly educate the public for the first five years of the epidemic.

The medical community, faced with caring for hundreds and soon thousands of AIDS patients, responded more appropriately to the epidemic. In particular, the country's highest-appointed public health figure, the late Surgeon General Everett Koop, became a staunch proponent for reducing transmission. However, politics severely impeded his efforts to adequately address the epidemic in the beginning [23]. Finally in 1986, only with President Reagan's approval, he published the *Surgeon General's Report on AIDS* and later took the unprecedented action of mailing AIDS information to every single household in America [24].

Unfortunately, the medical community's research and educational efforts translated into neither prompt national acknowledgement of AIDS, greater understanding of risk behaviors, nor a reduction in denial. Beginning in 1983, groundbreaking studies began presenting evidence for the heterosexual and perinatal transmission of AIDS [25]. These studies suggested that the virus could spread not just from males to females but from *females to males*. People refused to believe that they could be just as at risk for HIV as these “other undesirable groups,” especially through “mainstream” heterosexual intercourse. A mountain of criticism, denial, and accusations ensued to refute this latest scientific finding. For example, a 1988 January issue of the magazine *Cosmopolitan* claimed that women were not at risk for infection when having unprotected intercourse with HIV-positive men [26]. Meanwhile, the virus kept spreading.

Insufficient government assistance and an insensitive public mobilized AIDS patients to come together and create strong advocacy groups. Many argue that this “proactive advocacy” movement contributed heavily, if not the most, to the country's eventually moving in a positive direction in addressing this epidemic [27]. The AIDS Coalition to Unleash Power and other groups fought for improved access to newly developed antiretroviral drugs, public education about HIV, national policies on AIDS, and other rights [27]. These efforts culminated in the public finally recognizing, regardless of sexual orientation, that they were in the midst of a massive epidemic, albeit 10 years after the initial 1981 AIDS report. To further emphasize this delay in acknowledgement, the United States reported more than 59,000 AIDS-related deaths by 1989, and the public still did not fully understand the severity of the epidemic [28]. The recent Ebola epidemic in West Africa jolted worldwide alarm after less than a thousand deaths [29].

The AIDS epidemic in the United States has changed. The government and general public recognize the formidable impact of AIDS and have mobilized efforts to reduce its incidence. In 2010, three decades after AIDS captured nationwide headlines, the United States finally created its first national comprehensive plan for this disease, called the National HIV/AIDS Strategy [30]. Unfortunately, there are still over a million HIV-positive people in the United States, one in seven is unaware of a positive serostatus, and the disease continues to carry enormous stigma [31].

## INDIA

In 1986, a female sex worker (FSW) in Chennai, Tamil Nadu, became the first patient in India diagnosed with AIDS [32]. At least 100 more FSWs with the disease surfaced the following year. As India had never reported AIDS cases before, many believed that outside visitors brought the virus to the country [32]. Soon after, injection drug users and men who have sex with men emerged as additional risk groups for the disease. While the United States shared these susceptible populations, transgender individuals and truck drivers were more prominent high-risk groups in India [33]. The former often participated in sex work for income, and the latter typically had multiple sexual partners across different cities and consequently passed the virus along to their wives.

Similar to the United States, the public in India berated AIDS patients [34]. The police violently attacked those having the disease, and other groups thought to have the disease were denounced. Public hospitals and medical professionals refused care to AIDS patients. Temples and houses of worship prohibited them from entering. Families ostracized infected members, believing they had acquired the virus due to shameful behavior, and sometimes denied their last rites after death. In India, the fear of this discrimination has been linked to significant reluctance to seek testing and treatment, which consequently assisted in spreading the virus [35].

Indian government officials responded negatively from the beginning. When the Tamil Nadu Parliament learned that their state had hosted India's first known AIDS case, they verbally and publicly reprimanded those involved with the discovery [32]. Another example of denunciation occurred in 1987. Researchers and medical professionals wanted to perform prospective studies on FSWs in several Indian cities [36]. Combating a new epidemic requires knowing who is susceptible, major risk factors for transmission, and other epidemiological characteristics. Unfortunately, the Indian government forbade this project [36]. Instead, officials announced that they would control the disease by limiting foreigners from entering the country. They believed, whether consciously or not, that the virus was an extrinsic pathogen brought into the country, and not associated with sex work, drug use, and other high-risk behaviors already present in India.

This mindset arguably contributed to regrettable initiatives aimed at controlling the epidemic. A notable example occurred in Maharashtra during the mid-1990s. The state's Women and Child Welfare director began circulating a directive demanding that all women above 12 years of age in orphanages and homeless shelters undergo mandatory HIV testing [37]. Infected girls were immediately placed in isolation at a medical institution and physicians treated them without consent. Many physicians, lawmakers, and ethicists challenged this directive, stating how it violated basic human rights [38,39]. The initiative nonetheless drove the epidemic further underground.

In 1992, incidence rates skyrocketed to embarrassingly high levels. Finally, the Indian Parliament responded by creating the National AIDS Control Organization (NACO) to spearhead India's comprehensive effort to prevent and control AIDS [38]. The first of NACO's three phases spanned seven years (1992–1999) and monitored HIV infection rates among high-risk populations. The second (1999–2006) and third (post-2006) phases involved implementing targeted interventions and preventive measures for high-risk populations, and expanding treatment, medical, and support services nationwide. Additionally, officials contacted the original investigators to begin the aforementioned prospective and epidemiological studies [36]. By 1996, India still harbored the greatest number of AIDS patients internationally [36].

Advocacy groups have had some of the largest impacts on education, treatment options, improving the understanding of high-risk behaviors, and the creation of support groups. A major accomplishment occurred in 2009. The Naz Foundation, a non-governmental organization, had challenged Section 377 of the Indian Penal Code as unconstitutional in 2001 [39]. This section declared homosexuality as an “unnatural” offense with penalties including “life imprisonment,” and dated back to when the British colonized India. After eight years, the New Delhi High Court ruled in the Naz Foundation's favor, thereby decriminalizing homosexuality. Unfortunately, this success only lasted a few years, as the Supreme Court of India overturned this judgment in 2013 [40].

As of this date, India, like the United States, has improved its response to the challenges of the epidemic. Numerous nonprofit organizations and advocacy groups have promoted screening and provided treatments, significantly slowing the transmission of the disease. NACO reports a “declining epidemic at the national level, though inter-state variations exist” [41]. Yearly HIV incidence has declined by 57% in the past decade [41]. Unfortunately, there are still 2.1 million people with HIV in India and, of these, it is unknown how many know that they are infected [42].

## LESSONS FROM THE EARLY AIDS PANDEMIC

The early AIDS epidemics in the United States and India share many remarkable similarities (Table 1) despite different cultures, political environments, and social structures. Analyzing these similarities allowed us to elucidate vital and generalizable lessons necessary for combating epidemics. Such lessons often illustrate the necessity of protecting basic human rights and highlight how different segments of society (i.e., government, the medical community, etc.), must respond during the early stages of an epidemic. Importantly, these lessons can inform complicated and ethical public health questions during future epidemics. Here we describe four key lessons.

**Table 1.** Early Responses and Characteristics of the AIDS Pandemic in the United States and India

Category	United States	India
**First identified patients**	Homosexual males	Female commercial sex workers
**Initial marginalized groups**	Homosexuals, Haitians, hemophiliacs, heroin (injection drug) users	Commercial sex workers, homosexuals, transgender individuals, truck drivers
**Key government responses**	President Reagan first publicly mentions AIDS in 1985, four years after the epidemic began Supported abstinence; contemplated banning HIV-positive immigrants from entering the U.S. Refused to endorse safer sex and contraception Discriminated against homosexuals	Government of Tamil Nadu verbally reprimands investigators who identified the first Indian person with AIDS Supported banning HIV-positive travelers from entering India, strict quarantine initiatives Culture prohibited an open and honest conversation about sexuality Continued laws criminalizing homosexuality

A pervasive theme that underscores the response to the epidemic by the general public and the government in both the United States and India is denial. Denial in accepting that the entire public was in the midst of a rapidly growing epidemic led to the delays in implementing public health measures, the marginalization of patients with AIDS and those at high risk, and the development of compulsory laws that only further fueled the spread of the virus. Accordingly, attempts to control an epidemic must first begin with government recognizing and acknowledging high-risk behaviors without judgment. Denying the presence of these behaviors promotes transmission of the pathogen and perpetuates harmful rumors. The early AIDS epidemics in the United States and India clearly display the disastrous consequences, the danger to patients, the inertia around beginning interventions, and various human rights violations that can occur when there is denial and stigmatization. Governments must initiate proactive campaigns to educate and destigmatize high-risk behaviors from the very beginning.

In the United States, the Surgeon General, officially the nonpartisan medical representative for the country, theoretically serves a key role by outlining recommendations for controlling an epidemic. However, this was not the case during the early years of the AIDS epidemic. The medical community's potential to address a national epidemic becomes severely impaired without governmental support. The Tamil Nadu Parliament's public shaming of the original Indian investigators further underscores this point. Societal perception and political forces simply overwhelmed early efforts by the medical community to curb transmission. Prompt interventions require a synergistic partnership between the government and the medical community.

Once the government and medical community work together, the next step requires implementing public health measures that are ethical and nondiscriminatory. When these initiatives fail to uphold the rights of patients and those working with patients, they become counterproductive in curbing transmission. Ill-conceived initiatives that deny these rights can drive the epidemic underground, spread inaccurate information about risk, and contribute to the already problematic discrimination and stigma experienced by infected individuals during an epidemic.

Finally, governments must actively protect the human rights of groups at high risk for acquiring the pathogen and provide them with adequate medical care. Such efforts will help to control an epidemic that likely will spread to the general population. But it is important to recognize that treating these persons only as a means to an end (i.e., the protection of others) strips these individuals of their dignity. All individuals are entitled to adequate and ethical medical care without prejudgment. Failure to respect this principle denies a basic human right and promotes physical and emotional harm, consequently fueling an epidemic.

## EBOLA AND FUTURE EPIDEMICS

We identify four key lessons for prompt, efficient, and adequate epidemic control: (1) authoritative officials must acknowledge the presence of high-risk behaviors and properly educate the public without stigmatizing groups of individuals; (2) the medical community and federal government must form and display a respectful and collaborative partnership to the public; (3) government and medical officials must collaborate to implement effective, yet civil, interventions for limiting transmission; and (4) these same officials must ensure that their interventions maintain the human rights of high-risk populations and of healthcare workers.

Regarding the Ebola epidemic in West Africa, the governments and medical communities of the affected countries have made tremendous strides. Public health officials identified several high-risk behaviors that contribute to the spread of Ebola. Some included caring for an infected person, handling of bodily fluids, or preparing the body of someone who has died from Ebola for cultural burial rituals. Unfortunately, fear and the media propagated false notions about how the virus can spread [43]. This required more diligent mechanisms for properly educating the public about how the virus can spread and ensuring that blame for the disease is not placed on any particular group.

Establishing a respectful and collaborative partnership between the medical community and government can be challenging. As an example, many politicians called for travel bans between the United States and West Africa and mandatory quarantine for all returning healthcare workers despite disapproval of these measures by the CDC and the National Institutes of Health [3]. Such policies send the wrong message to healthcare workers and further stigmatize the infected. Furthermore, these views reinforced the persistent stigmatization of West Africans, potentially derailing on-the-ground initiatives to hinder spread of the virus. Clearer communication and better collaboration between the medical community and the government are needed to inform and protect the general public.

The final two lessons require implementing civil interventions that are effective in limiting transmission, while maintaining the rights of high-risk populations and of healthcare workers. Prompt, efficient interventions, such as appropriate and ethical quarantining, close monitoring, contact tracing, and distribution of treatments have halted the epidemic in several countries, including Nigeria, Senegal, and Mali [44]. More recently, the CDC declared Liberia free of Ebola virus transmission as of May 9, 2015, Sierra Leone as of November 7, 2015, and Guinea as of December 29, 2015. At least 11,000 people have died from the virus in these three countries [44].

To continue making progress, we must learn from our history. We cannot afford to repeat past mistakes from previous epidemics when battling the inevitable epidemics of the future.
